# Psychometric properties of observational tools for identifying motor difficulties – a systematic review

**DOI:** 10.1186/s12887-019-1657-6

**Published:** 2019-09-07

**Authors:** P. Asunta, H. Viholainen, T. Ahonen, P. Rintala

**Affiliations:** 1 Faculty of Sport and Health Science, University of Jyväskylä, P.O. Box 35, FI-40014 Jyväskylä, Finland; 2 Faculty of Educational Sciences and Psychology, University of Jyväskylä, P.O. Box 35, FI-40014 Jyväskylä, Finland

**Keywords:** Developmental coordination disorder, Questionnaire, Assessment, Psychometric properties

## Abstract

**Background:**

Early identification of children with motor difficulties, such as developmental coordination disorder (DCD), is essential. At present only a fraction of children with DCD are identified. The purpose of the study was to systematically review the literature from 1994 to 2017 on observational screening tools and to evaluate the validity, reliability and usability of the questionnaires used.

**Methods:**

The review of the literature was conducted to synthesize the data from five electronic databases for children aged 6–12 years. The following databases were searched: Academic search Elite (EBSCO), ERIC (ProQuest), MEDLINE (Ovid), PsycINFO (ProQuest), and SPORTDiscus with Full Text (EBSCO). The studies meeting our inclusion criteria were analyzed to assess the psychometric properties and feasibility of the measures.

**Results:**

The literature search retrieved 1907 potentially relevant publications. The final number of studies that met the inclusion criteria of our systematic review was 45. There were 11 questionnaires for parents, teachers and children. None of the questionnaires was valid for population-based screening as the only measurement tool.

**Conclusions:**

There are many challenges in using initial screening tools to identify children with motor difficulties. Nevertheless, many promising questionnaires are being developed that can provide information on functional skills and limitations across a variety of tasks and settings in the daily lives of children with DCD. The review provides much needed information about the current scales used in many clinical, educational and research settings. Implications for assessing psychometric properties of the developed questionnaires and further research are discussed.

**Trial registration:**

PROSPERO, CRD42018087532.

**Electronic supplementary material:**

The online version of this article (10.1186/s12887-019-1657-6) contains supplementary material, which is available to authorized users.

## Introduction

Developmental coordination disorder (DCD) has been discussed for 20 years, at present only a fraction of children with DCD are identified [1]. DCD is still poorly understood by many healthcare and education professionals [2], although, DCD affects 5–6% of school-age children. It is characterized by a major impairment of motor coordination and typically has a significant negative impact on the performance of everyday activities or academic achievement [3].

Early assessment and identification of children at risk for DCD are important in order to avoid these secondary physical, cognitive, language, and social–emotional manifestations of the disorder [4, 5]. There is considerable evidence that difficulties to acquire and execute motor skills can lead to secondary problems, such as poor self-esteem and other psychosocial issues [6, 7] and physical health problems [8]. Furthermore, DCD is commonly associated with other developmental disorders [9], such as attention deficit/hyperactivity disorder (ADHD) [10, 11], learning disabilities such as dyslexia and specific language impairment (SLI) [12], and autism and associated psychosocial impairments [13, 14]. However, identification of DCD is difficult especially in school context because of DCD’s heterogeneity in severity and comorbidity and its appearance in the area of fine and/or gross motor skills.

Up to now, the greatest emphasis has been on diagnostic screening. Especially, in the field of DCD, the goal has been to identify those with movement difficulties [15]. Along with home, the school environment is a place where children spend a lot of time; therefore, teachers perceive the child’s performance in everyday activities and academic learning, which is one of the diagnostic criteria of DCD [3]. There are also studies that emphasize the importance of involving teachers in DCD screening [16, 17]. Moreover, providing teachers with an easy-to-use method for identifying problems in motor learning could support them in their quest to enhance all children’s motor learning. Practical tools for teachers are needed, because it has been found that teachers are more likely to recognize motor problems if nondisruptive behavior is present [18]. This is alarming, since we know the comorbidity with DCD and other psychosocial difficulties [11, 19, 20]. However, we did not limit our interest strictly to school teachers, as our focus of screening tools was context free.

Few observational tools for teachers, parents, children and nurses to identify children with motor learning problems have been developed. Those checklist-type tools have been extensively used both in research and non-research settings in the field of DCD [15]. Barnett [15] has highlighted that further studies are needed to establish the utility of each of these instruments to accurately identify children with DCD.

Therefore, we were interested in evaluating which of the developed questionnaires could be feasible, valid, and reliable for further development as cultural adaptations, which enable exchange of information and facilitate collaboration between countries and which furthermore are cheap and fast [21]. There being no replicable studies available, we conducted our own comprehensive systematic review. The specific aim of the systematic review was: (1) to investigate the questionnaire-based (paper-pencil) identification tools for psychometric properties and (2) to describe the usability in identifying motor difficulties in primary school-age children (6–12-years old) in different environments. We use words ‘identifying’ and ‘screening’ as synonyms, though there is a small difference between them. Identifying is more suitable in educational approach and screening in medical and research settings.

Currently, there is no gold standard tool to assess children with DCD [7]. Many instruments are available to investigate motor ability in children [5]. In order to measure movement competence, a wider range of test batteries is recommended [22], as well as a multi-stage approach. In a multi-stage approach a preliminary screening is usually carried out by questionnaire-based observational tools, which provide economical and effective first-step assessment [23], and the results can be followed or confirmed by standardized tests [24–26].

Despite the advisability of early assessment and identification [16, 24, 26] and the development of many screening instruments, there are no gold standard observational tools available either. Indeed, although the disorder is so common, basic information about feasible and valid observational questionnaires for identifying problems in motor skill acquisition, which is one the most important criteria of DCD, is still lacking. Furthermore, it is uncertain who might be the most reliable and valid person to make qualitative observations: teacher, parent, or the child him/herself. Green and Wilson [27] have suggested that parents and children can assist in the screening process, because their judgments about movement difficulties are valid. However, it has been postulated that parents and teachers often over-refer the problems [28]. In contrast, parents’ information is arguably essential to determine, whether the child’s motor impairment is actually impacting on everyday activity like self-care skills (e.g., washing and dressing), Along with home, the school environment is a place where children spend a lot of time; therefore, teachers perceive the child’s performance in everyday activities and academic learning. The screening instruments in home and school settings can be usefully applied to the assessment of criterion B, to obtain information on the range of everyday life skills (ADL) that the child finds difficult, which is one of the diagnostic criteria of DCD [3, 29].

Psychometric properties refer to the validity and reliability of the measurement tool. Before being able to state that a questionnaire has excellent psychometric properties, meaning a scale is both reliable and valid, it must be evaluated extensively [30].

Information on usability can be gathered and described on both the literature and the experience of people using experts, user interviews and statistics. For practicability the following features can be evaluated: price, availability / usage restrictions, education needed, time requirements, ambiguity and ease of interpretation of results (including availability of reference values).

Many studies have underlined the challenges of using initial observational screening tools to identify children with DCD in population-based samples [31, 32]. In clinical studies, the concurrent validity (sensitivity and specificity rates) are somewhat better than in population-based studies, but they are still not acceptable [32]. Screening tools have been shown to have the ability to identify true cases of DCD (sensitivity) when it is present but infrequently the ability to exclude DCD when it is absent, in other words correctly to identify children without DCD (specificity) [29]. However, good sensitivity (> 80%) is more preferable in population screening than high specificity (> 90%) in order to identify all children at risk [25, 32]. Sensitivity has been found to be generally weaker in population based data sets than in clinical-referred samples [32].

## Method

### Protocol

Details of the protocol for this systematic literature review was registered with the international database of prospectively registered systematic reviews, PROSPERO, and given the registration number CRD42018087532 (can be retrieved at www.crd.york.ac.uk/PROSPERO/display_record.asp?ID=CRD42018087532.). Our search strategy utilized and combined the following main areas of keywords and synonyms. The terms were chosen according to the study questions and from those found in the literature on DCD studies, as indicated in our preliminary search. We had two groups of words (A and B). The words / key terms in group A were synonyms for DCD: clumsy children, developmental coordination disorder (DCD), probable DCD, motor skills disorder, minimal brain dysfunction, dyspraxia, movement disorders, motor problems, motor difficulties, motor learning difficulties, incoordination, and motor delay. The terms in group B described observational measurement tool: screening, screening tool, questionnaire, and checklist. To be considered for inclusion in the review, the title or abstract of the study had to contain at least one term from both of the groups (A and B).

The following five electronic databases were searched for the review: Academic search Elite (EBSCO), ERIC (ProQuest), MEDLINE (Ovid), PsycINFO (ProQuest), and SPORTDiscus with Full Text (EBSCO). In addition, we conducted searches in Google Scholar to retrieve supplementary information. Information was also sought manually, for example among the references in the identified publications, and EACD recommendations [33] were reviewed. Colleagues in the field were also consulted.

The search, which was designed to be inclusive and accurate, followed research guidelines [34]. Database-controlled vocabulary (Thesaurus) was used whenever possible. The terms used were tailored for each database. Full details of the searches can be found in Additional file 1.

Studies were included if the following criteria were met: (1) published in a peer-reviewed journal; (2) published between 1994 and 2017; (3) containing at least one term from both keyword groups (A and B); (4) relating to children aged six to 12 years (or mean ≥ 6); (5) English language; (6) observational questionnaire (paper-pencil instrument).

Studies were excluded: (1) related only to clinical assessment screening tests, because our interest was in finding questionnaire-based, short, and easy-to-use methods for identifying problems in motor learning; (2) they fell outside the diagnostic exclusion criteria of DCD according to DSM-V [3], such as neurologic disorders, other specific learning disabilities, or intellectual disability.

In the first stage of the screening process, the studies were considered based on their titles and abstracts. The second stage was approval on the basis of the full text. Manually found articles were included in the full text screening stage. Two independent reviewers (PA and HV at the level of titles and abstracts, and PA and PR at full text level) screened and selected articles at each stage of the selection process and checked the differences between the accepted titles, abstracts, and full texts. Where there was disagreement, reviewers discussed the issue until they reached a consensus. Consistency between the two authors before consensus discussions varied from 94% at the abstract level to 92% at the full text level.

### Evidence synthesis and quality assessment

Studies that were selected, having met our inclusion criteria, were reviewed to collect descriptive psychometric information. They were divided according to their terms of measurement, aim, age, scope/population, and psychometric properties (see Additional file 2). The quality of the selected articles was evaluated based on the Grading of Recommendations Assessment, Development, and Evaluation (GRADE) methodology. GRADE classifies the quality of evidence as high, moderate, low, or very low [35–37]. Since this method is primarily intended to evaluate interventions and diagnostic tools, we modified the GRADE criteria (see Table 1). For example randomized trials without important limitations provide high quality evidence and observational studies without special strengths or important limitations provide low quality evidence. Factors that reduce or increase the level of evidence 1 or 2 levels, are described in Table 1. For instance, if the sample selection is well described, sample size is large or very good representativeness of the population and we think that the data has been analyzed with relevant statistical tests and quality of results are good, it is possible to reach the highest level of evidence.

**Table 1:** Level of evidence (GRADE) adapted from Guatt et al. [36] and Horvath [37]

Factors that reduce or increase the level of evidence	GRADE
Further research is very unlikely to change our confidence in the estimate of effect; Very good quality of the results (validity and reliability measures > 0.8); Well described sample selection; Large sample size (*n* > 100 /for each group) or very good representativeness of the population that was intended to be sampled Confirmatory data analysis and relevant statistical test(s) Large magnitude effect;	1 (high)
Further research is likely to have an important impact on our confidence in the estimate of effect and may change the estimate; Good quality of the results (validity and reliability measures > 0.6); Adequate sample size (n = 30–100 / for each group) or good representativeness of the population that was intended to be sampled;	2 (moderate)
Further research is very likely to change our confidence in the estimate of effect; Moderate quality of the results (validity and reliability measures > 0.4); Small sample size (*n* < 30 / for each group) or weak representativeness of the population that was intended to be sampled Wide confidence intervals for estimates of test accuracy, or true and false positive/negative rates; Unexplained inconsistency in sensitivity, specificity or likelihood ratios;	3 (low)
Any estimate of effect is very uncertain; Evidence from expert committee report or experts; Sample size or selection not described; Wide confidence intervals for estimates of test accuracy, or true and false positive/negative rates; Unexplained inconsistency in sensitivity, specificity or likelihood ratios;	4 (very low)

In a modern view “validity is ensuring the appropriateness of an inference or decision made from measurement” [38]. Further, validity can be thought to be a characteristic of the inferences made based on the results obtained using the questionnaire or measurement tool [39]. Continuous validity evaluation of the developed methods is essential and should be viewed as a unified concept [38, 39].

We looked for different aspects of empirical validity evidence, including concurrent, predictive, construct, known group/discriminative, convergent, cross-cultural, and face validity. *Concurrent validity* relates to how well a measure compares to a well-established test, which is often a standardized “gold standard” test, and the evidence is obtained about the same time as the target measurement. *Predictive validity* is often described in terms of sensitivity and specificity. Sensitivity is the ability for a measurement to detect someone with a condition (e.g., a child with DCD). Specificity refers to the ability to correctly identify those persons without a condition (e.g., children developing typically). The required standard of sensitivity is > 80% and of specificity > 90% [3]. *Construct validity* is relevant to the perceived overall validity of the measurement. It is defined as the theoretical basis for using the measurement, and the methods used are often factor analysis. *Known group validity* examines whether a test distinguishes between a group of individuals known to have a DCD and a group who are developing typically. *Discriminative validity* verifies that measures or tests that should not be related are in reality not related. *Convergent validity* refers to the degree to which two measures of constructs that theoretically should be related are in reality related. Known group and discriminative validity and convergent validity are all considered subcategories of construct validity. *Cross-cultural validity* applies when questionnaires have been translated into different cultures and languages. Validity can be explored by comparison of score level attributes or measurement constructs between the original and adapted versions: Does the scale work in the same way in a different population (measurement invariance and differential item functioning)? *Face validity* refers to the extent to which one or more individuals subjectively think that a questionnaire appears to cover the concept it purports to measure.

Reliability is the overall consistency of a measure, describing the extent to which a measure is stable when repeated under consistent conditions. First, *test–retest reliability* refers to the relative stability of the assessment over time, assessing the degree to which the measurement tool scores are consistent from one test administration to the next. Second, *inter-rater reliability* assesses the degree of agreement between two raters. Third, *internal consistency* assesses how well the items in the questionnaire measure the same construct. Measures of 0.80 or above are considered excellent, and the minimum acceptable value is 0.70 [40].

## Results

**Fig. 1:**
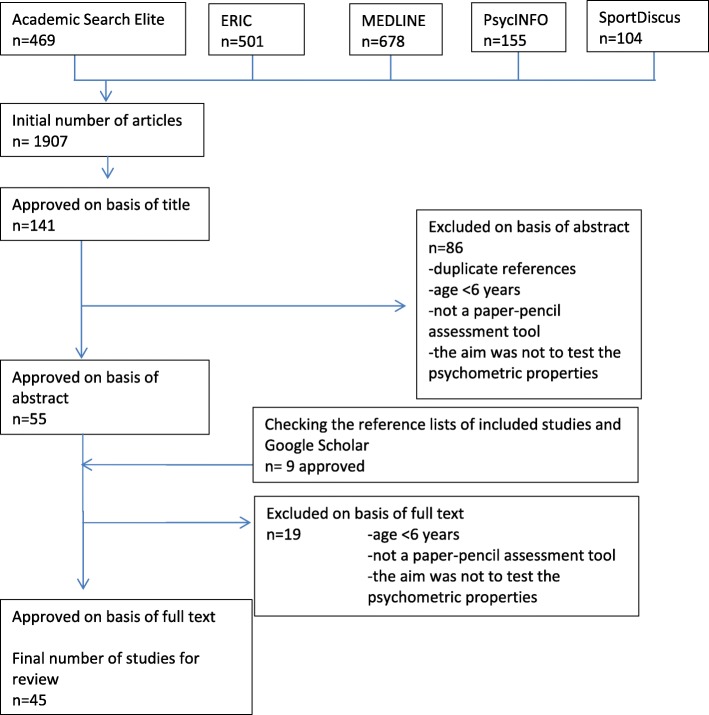
Flow chart of article selection

The literature search retrieved 1907 potentially relevant publications (see Additional file 1). Of these, 1766 studies failed to meet the inclusion criteria, and 141 eligibility studies were selected. After additional searches and exclusions (Fig. 1), the final number of studies that met the inclusion criteria of our systematic review was 45. Altogether, 11 different questionnaire-based screening tools were found, originating from 17 different countries from every continent. Six questionnaires were intended for teachers, five for parents and one for children (see Tables 2, 3 and 4).

**Table 2:** Descriptive characteristics of observational questionnaires completed by teachers

Measure	Country	Age range	Studies involved	Conclusions and main findings1
ChAS-T	Israel	4–8 yrs.	Rosenblum [58]	*Strengths:* -Good item consistency and concurrent validity -Distinguishes between children with and without DCD *To be developed:* -Larger samples and wider age range (validity and reliability studied only in age range of 5–6.5 years) -Gender difference not studied -No sensitivity or specificity scores -Neither intra-rater nor test–retest reliability results
Checklist	UK	school-age children	Dussart [71]	*Strength:* -The first screening instrument developed for teachers in the normal school population *To be developed:* -Sample selection and size not described -No reliability information -Validity studied only superficially -Many false positives
GMRS	Netherlands	3–7 yrs.	Netelenbos [61]	*Strength:* -Good reliability *To be developed:* -No sensitivity and specificity scores
M-ABC-C / M-ABC-2-C	UK	5.4–15.6 yrs.	Capistrano et al. [72]; De Milander [73]; Green et al. [67]; Junaid et al. [63]; Piek & Edwards [43]; Schoemaker et al. [25]; Schoemaker et al. [44]; Wright et al. [45]; Wright & Sugden [74]	*Strengths:* -Some good test–retest reliability scores -Translated in many countries *To be developed:* -Too long and time-consuming -Very low sensitivity: none of the studies met the required criteria -Inter-rater reliability not studied
MOQ-T	Netherlands	5–11 yrs.	Asunta et al. [41]; Giofre et al. [42]; Schoemaker et al. [62]	*Strengths:* - Good construct validity - Sensitivity met the criteria - Good discriminant validity and concurrent validity - High internal consistency - Good sample sizes - Both population and clinical referred samples - Fast to fill, usability good *To be developed:* - Specificity is slightly too low - Inter-rater and test–retest reliability not studied
TEAF	Canada	6–11 yrs.	Faught et al. [91]; Engel-Yeger et al. [16]; Rosenblum & Engel-Yeger [47]	*Strengths:* - Sensitivity met the criteria - Predicts participation preference *To be developed:* - Specificity is slightly too low - No inter-rater or test–retest reliability scores

*Note*. ChAS-T= Children Activity Scale for Teachers; GMRS= Gross Motor Rating Scale; M-ABC-C= Movement Assessment Battery for Children Checklist; M-ABC-2-C= Movement Assessment Battery for Children Checklist – Second Edition; MOQ-T= Motor Observation Questionnaire for Teachers; TEAF= Teacher Estimation of Activity Forms

^1^Conclusions and main findings are recapitulated by authors. Good sensitivity (>80%), high specificity (>90%)

**Table 3:** Descriptive characteristics of observational questionnaires filled in by parents

Measure	Country	Age range	Studies involved	Conclusions and main findings^1^
CAMP	Hong Kong	5–10 yrs.	Tsang et al. [59, 87]	*Strengths:* - A promising measure - Distinguishes between children with DCD and TD children - Good test–retest reliability *To be developed:* - Predictive validity, usability and inter-rater -reliability not studied
CBCL	Australia	3.9–14.10 yrs.	Piek et al. [7]	*Strengths:* - Some of the items bore a relationship to motor ability, but they should not be used to screen DCD children *To be developed:* - Discrimination accuracy and sensitivity are poor - No reliability studies done
ChAS-P	Israel	4–8 yrs.	Rosenblum [58]	*Strengths:* - Good item consistency and concurrent validity - Distinguishes between children with and without DCD *To be developed:* - Small sample size - validity and reliability studied only in the age range of 5–6.5 years -Gender difference not studied - No sensitivity or specificity rates, neither intra-rater nor inter-rater reliability results
DCDQ	Canada	5–15 yrs.	Cairney et al. [66]; Caravele et al. [75]; Caravale et al. [54]; Civetta & Hillier [76] Girish et al. [77]; Green et al. [67]; De Milander et al. [89]; Kennedy-Behr et al. [78]; Loh et al. [79]; Martini et al. [48]; Missiuna et al. [31]; Miyachi et al. [80];	- Most studied and evaluated questionnaire - A valid clinical tool, but not for population-based screening *To be developed:* - No inter-rater reliability results - No face validity
DCDDailyQ	Netherland	5–8 yrs.	Van der Linde et al. [53]	*Strengths*: - Excellent discriminant validity and predictive validity *To be developed*: - No reliability results - Usability descriptions and evaluation

*Note*. CAMP= Caregiver Assessment of Movement Participation; CBCL= Child Behavior Checklist; ChAS-P= Children Activity Scales for Parents; DCDQ= Developmental Coordination Disorder Questionnaire

^1^Conclusions and main findings are recapitulated by authors. Good sensitivity (>80%), high specificity (>90%)

**Table 4:** Descriptive characteristics of approved studies completed by children

Measure	Country	Age Range	Studies involved	Conclusions and main findings1
Children				
CSAPPA	Canada	9–16 yrs	Cairney et al. [24]; Hay et al. [90]	*Strengths:*
				-A promising screening instrument for DCD -Specificity low in population-based sample -Gives important information on child’s perception *To be developed:* -Reliability and usability not studied

*Note. CSAPPA* Children’s Self-Perceptions of Adequacy in and Predilection for Physical Activity Scale

1Conclusions and main findings are recapitulated by authors. Good sensitivity (> 80%), high specificity (> 90%)

Additional file 2 provides a summary of the characteristics of the studies included in the review. The quality of evidence, the GRADE evaluation and psychometric properties in reviewed articles can be found in Table 5. Cross-cultural adaptations, in which reliability and/or validity was investigated in a different country from the one in which the original questionnaire was developed was examined in most of the studies (*n* = 26; 58%).

**Table 5:** Psychometric properties of the questionnaires

	Usability described	Methodological quality										Quality of the evidence	
	1	2	3	4	5	6	7	8	9	10	11	Sum	GRADE
1. Questionnaires for parents													
1.1 CAMP													
Tsang et al. [59]		+	+	+	+			+	+			6	2
Tsang et al. [87]		+			+			+		+		4	1
1.2 CBCL													
Piek et al. [7]		+	+	+	+							4	2
1.3 ChAS-P/T													
Rosenblum [58]	+	+		+	+			+	+			6	2
1.4 DCDQ													
Rivard et al. [84]				+	+							2	2
Patel & Gabbard [83]	+			+			+		+	+		5	2
Nowak et al. [45]		+	+		+		+		+	+		6	2
Montoro et al. [81]				+			+					1	3
Miyachi et al. [80]					+	+	+					2	3
DE Milander et al. [89]		+	+			+	+					4	2
Cairney et al. [66]				+		+			+			3	2
Caravele et al. [75]			+		+		+		+	+		5	2
Caravale et al. [54]		+	+	+			+		+	+		6	2
Civetta & Hillier [76]		+	+	+	+	+	+		+			7	1
Girish et al. [77]	+			+			+		+	+		5	2
Green et al. [27]		+	+		+							3	2
Kennedy-Behr et al. [78]	+	+	+	+	+		+		+			7	2
Loh et al. [79]		+	+		+	+	+					5	2
Martini et al. [48]	+	+	+	+	+	+	+		+	+		9	2
Nakai et al. [57]				+		+	+		+			4	2
Prado et al. [82]	+		+		+		+		+	+		6	3
Schoemaker et al. [4]		+	+	+	+		+		+			6	1
Tseng et al. [85]		+	+	+	+		+		+	+		7	1
Wilson et al. [86]	+	+		+	+	+			+			6	2
Wilson et al. [64]	+	+	+	+					+			5	2
Ray-Kaeser et al. [56]	+						+					2	2
1.5 DCDDailyQ													
Van der Linde [53]		+	+	+	+	+		+	+			7	1
2. Questionnaires for teachers													
2.1 ChAS-P/T													
Rosenblum [58]	+	+		+	+			+	+			6	2
2.2 Checklist													
Dussart [71]		+			+							2	4
2.3 GMRS													
Netelenbos et al. [61]		+	+	+		+			+	+	+	7	2
2.4 M-ABC-C													
Capistrano et al. [72]						+						1	4
De Milander [73]		+	+									2	2
Green et al. [67]		+	+		+							3	2
Junaid et al. [63]		+	+							+		3	2
Piek & Edwards [43]	+		+				+					3	2
Schoemaker et al. [25]		+	+	+	+		+		+			6	2
Schoemaker et al. [44]	+	+	+	+	+	+	+		+			8	1
Wright et al. [45]	+	+					+			+		4	2
Wright & Sugden [74]	+	+					+			+		4	2
2.5 MOQ-T													
Asunta et al. [41]	+	+	+	+			+		+			6	1
Giofre et al. [42]				+	+	+	+		+			5	1
Schoemaker et al. [62]		+	+	+	+	+			+			6	1
2.6 TEAF													
Engel-Yeger et al. [16]		+			+	+	+		+			4	2
Rosenblum & Engel-Yeger [46]		+	+	+	+		+		+			5	2
Faught et al. [88]		+	+	+	+	+			+			6	1
3.Questionnaire for children													
3.1 CSAPPA													
Cairney et al. [24]			+		+						2	2	
Hay et al. [90]			+		+					2	2		

*Note*. 1 = usability described; 2 = concurrent validity; 3 = predictive validity; 4 = construct validity; 5 = known group validity/ discriminative validity; 6 = convergent validity; 7 = cross cultural validity; 8 = face validity; 9 = internal consistency; 10 = test-retest reliability; 11 = inter-rater reliability; SUM = the number of usability, validity and reliability assessment, not equivalent; GRADE criteria (1 = high - 4 = low)

There were six tools intended for teachers’ use, for children in the age range of 3–12 years. Table 2 presents the descriptive characteristics. Four observational questionnaires were for parents suitable for children aged 3.9 to 15.6 years. The descriptive characteristics of the tools completed by parents are shown in Table 3. Children’s Self-Perceptions of Adequacy in and Predilection for Physical Activity (CSAPPA) for children was the only measurement in that target group, which was aimed for children in the age range of 9–16 years. Its descriptive characteristics are summarized in Table 4.

The Developmental Coordination Disorder Questionnaire (DCDQ), developed in Canada, had the most cultural adaptations in different countries and it has relatively more psychometric testing than the other tools included in this review. However, there are still some developmental needs. The inter-rater reliability and face validity has not been studied. Other cultural adaptations had MOQ-T [41, 42], MABC-2-C [25, 43–45] and TEAF [16, 46].

### Data synthesis

The heterogeneity of measurement tools and study design makes comparison of screening tools very challenging. We found different kinds of samples: clinical-referred and population-based. In addition, all of the studies reviewed in this paper did not use DCD-term. But they determined children with motor coordination problems for the most part at or below the 5th or 15th percentile, which is one of the four and arguably the most important criteria of DCD [47]. Further, the studies used different measurement tools as a “gold standard” and different cut-offs to distinguish children with DCD from children developing typically; therefore, comparisons of the psychometric properties of the questionnaires are complex. Because of the difficulty of comparing the questionnaires, we recorded the advantages (strengths) and developmental needs separately from each questionnaire in Tables 2, 3 and 4. In the Additional file 2 detailed psychometric properties of the studies are described. Based on the quality evaluation (GRADE), we have confidence in those questionnaires that have been properly planned (study selection, sample, methods) and implemented carefully and of which validity and reliability criteria are acceptable. Overall, the quality of the studies was relatively good. Ten of the studies included met the highest criteria in the quality of evidence classification system (GRADE, Table 5).

### Synthesis of psychometric properties of the questionnaires

Outcomes of psychometric properties represented in these studies were usability (*n* = 14), concurrent validity (*n* = 31), predictive validity (*n* = 29), construct validity (*n* = 27), known group validity/discriminative validity (*n* = 30), convergent validity (*n* = 16), cross-cultural validity (*n* = 25), face validity (*n* = 5), internal consistency (*n* = 28), test–retest reliability (*n* = 12), and inter-rater reliability (*n* = 1). As shown in the above and in Table 4, reliability, other than internal consistency of measures, was examined in very few studies. In summary, the inter-rater reliability and face validity were examined the least, and concurrent and discriminative validity was investigated the most. The greatest variability in terms of considering reliability and validity were the studies of Martini et al. [48] and Schoemaker et al. [44].

Convergent validity between two observational questionnaires varied from 0.16 to 0.64, and concurrent validity between a questionnaire and a motor/screening test, correlation outcomes ranged between 0.037 and 0.76. The good concurrent validity values were found when DCDQ-PL was compared to KTK-test (r = 0.73) and the TEAF to MABC test (r = 0.76). The most frequently used test to evaluate the concurrent or predictive validity with the questionnaire was MABC or MABC-2 (60%) [49, 50]. Clearly less used were BOTM or BOTM-SF (8,9%) [51], and MAND (4.4%) [52]. Other measures, like KTK, and TGMD, were used under 2,3% of the cases.

Sensitivity varied in clinical referred samples between 29 and 88% and in population-based samples from 17 to 88%. The specificity of the questionnaires ranged from 27 to 98% in population samples and from 19 to 95% in referred/clinic samples. Just one questionnaire, DCDDailyQ [53] reached the desired standard of predictive validity in population-based sample (sensitivity 88% and specificity 92%; AUC .961). In clinical samples, only one measure, DCDQ-Italian [54], was adequate (sensitivity 88%, specificity 96%), but the sample size was too small for this measure to be recommended for the present purpose.

### Synthesis of the usability of the questionnaires

Usability of the questionnaire was described only in 31% of the studies (see Table 5). In these studies, the most descriptions dealt with how much time evaluating requires, or how many questions / items are included in the measure. Whether users understand the questions, were explored only in a few studies. There was no study in which usability had been evaluated accurately or comprehensively. However, the ChAS-P/T and MOQ-T-FI questionnaires have well described usability.

## Discussion

This review evaluated 45 relevant studies and 11 observational tools for screening DCD. Overall, in many of these questionnaires, the psychometric properties and/ or feasibility was not extensively studied.

Validity evidence of a measurement tool cannot be generalized to all situations or with different attributes of population [38], therefore continuous validity and reliability evaluation of the developed methods is urgent. The translations and cross-cultural validations should be undertaken with the most stringent research design (see guidelines Beaton et al. [55]). Cognitive interviewing, which was used by Ray-Keaser et al. [56], seemed for example to be highly competent and quality approach to evaluate the cultural validity and usability of the measure.

The first step in identifying children with DCD is to be clear about the purpose of the assessment and then choose a test/tool that has been validated in that purpose [23]. Barnett [29] suggested also that selection of assessment tools to identify children with DCD should be justified and thought carefully. The selection of observational tools for children with motor difficulties will depend on their intended purpose: identification (i.e. educational settings), screening (i.e. health care), prediction, or evaluation (e.g. intervention). Many studies in this review claimed that they were appropriate for more than one purpose or in different samples. However, a measurement tool cannot be recommended if there is a lack of evidence about its psychometric properties. Therefore, it is important to be skeptical about the conclusions in some studies, because some did not have validity or reliability results that met the criteria, the sample size was too small, or the age range was too narrow [57–59]. Missiuna et al. [31] underlines also that assessors need to determine whether the level of reliability is suitable for their particular needs, for example in the particular age groups.

We recommend collecting information about the child’s everyday life multiprofessionally and in different environments, because motor skills are often changing in diverse situations. Also in clinical practice, we would recommend using more than one observational tool to give information on motor skill difficulties in different ecological environments, this being one of the criteria in DSM-V [3] for the diagnosis of DCD.

Both reliability and validity studies should always add descriptions of the raters’ background, expertise and prior training with these questionnaires. Appropriate training of raters could minimize measurement error. It is shown that the validity will improve if the observer gets sufficient information about DCD and/or the screening tool [60]. Because training and information are affecting the results, we recommend that future studies should report precisely have the assessors been trained to use the measure or given information about the motor learning problems, like DCD.

According to diagnostic criteria of DCD, motor problems affect academic achievement, leisure, and play. Based on our review for teachers there are 6 tools which could be used to evaluate this issue. Nevertheless, our study shows that in many reviewed studies teacher ratings of motor skills suffer from low concurrent validity, as had been showed previously as well [61]. However, teachers’ opinions could provide further confirmation of the children’s difficulties [31]. Besides, there are some high correlations with standardized test: ChAS-T and MABC r = 0.75 [58] and MOQ-T and MABC r = .57 [62]. The physical education (PE) teachers were more able to detect motor learning problems than the classroom teachers [43, 63]. Most of the studies in which questionnaires were intended for use by teachers, lacked information on whether the teachers also teach physical education. Unfortunately, this information was missing from most of the reviewed studies.

Parents can be used to help screening children with DCD. Parents’ opinions have been found to correlate better with standardized clinical tests: e.g. concurrent validity between MABC and DCDQ r = .55 [64] and correlation between DCDQ and KTK r = 0.726 [65], but there has been found just moderate correlations with childrens’ options [66, 67].

The concurrent and predictive validities for some assessments were calculated based on judgements by the same persons, or assessments were carried out with different standardized tests. These kind of differences and variability make exact comparisons impossible. However, the low concurrent validity that was present in almost every study may be due to a difference between the nature of the activities assessed by the observational tool in real life and the standardized motor tests such as the MABC-2 [61, 67].

Predictive validity was higher in clinic-referred samples than in population-based samples. Some studies have been attempting to overcome the low sensitivity in population-based screening by implementing two-tier referral systems [31, 68]. However, low specificity (many false positives) is not such a notable concern in the school context, where assessment and support are closely linked to each other, and where the extent of support is based on recurrent assessments. Besides, in the educational context, when support is given by classroom or PE teachers or nursery teachers, extra physical activity and support for the children identified as false positives cause no harm and do not stigmatize them. For the identified children, no further assessment is necessary if support in the educational environment is deemed to be helpful. Therefore, high sensitivity is the most important issue in educational settings. However, in healthcare screening, a large number of false positives is a major challenge, because of the cost effectiveness of providing support.

Questionnaires could be used also to give information on how motor impairments are affecting children in their daily activities and in academic learning. Therefore, observational questionnaires may be useful in clinical settings and clinic-referred samples to gain a wider picture of a child’s motor ability in the school or at home. As things stand at present, none of the observational screening tools in this review could be recommended on its own for health screening of DCD. However, many of the tools can assist in the diagnosis of DCD. Multiple assessments and measurement tools are recommended to give information in different aspects of motor function; thus, it is important to develop and investigate such screening tools further. Our review reflects some limitation of the studies included. With few exceptions [26, 56, 62, 65] the study sizes were relatively small.

There are some limitations in this study as well. First, it is possible that some tools remained outside of this review, because we wanted to limit the search to school-age children. Second, our study was also restricted to literature in English, and most of the articles were published in Europe, North America, and Australia. Accordingly, some potential international publications could have been missed (see [69]: language China: [70]: language Persian). However, this review suggests that future research should focus on the validation process for the developed measures. Also, a systematic review should be carried out in the whole age range, especially in the early years and for adolescents and adults. A variety of different statistical measures were reported in this review to assess the psychometric properties. The implications or future research would be to evaluate those statistical methods used. In addition, to improve reporting quality of future studies, we recommend authors to justify the relevant statistical test(s).

## Conclusion

Many tools have been developed to help in identification and screening for motor difficulties, such as DCD. The selection of observational tools for children with motor difficulties will depend on their intended purpose: identification, screening, prediction, or evaluation. As follows, in many cases, the assessment needs to be multi-faceted and multi-professional. Overall, this study shows that there is no assessment tool, which can be used in population-based screening alone, because all those reviewed have low sensitivity or specificity or only superficially assessed reliability. In the future, psychometric property testing should be improved by addressing rater qualification and usability descriptions. In addition, the stability (test-retest reliability) and homogeneity (inter-rater reliability) should be evaluated more when assessing the psychometric properties of a questionnaire. The accurate descriptions about the usability of the questionnaires should not be forgotten either.

## Additional files

Additional file 1: Search strategy. (DOCX 14 kb)

Additional file 2: Psychometric properties of included studies. (DOCX 57 kb)

## Data Availability

All data generated or analysed during this study are included in this published article [and its supplementary information files].

## Abbreviations

ADBS: The Australian Disruptive Behaviors Scale
ADHD: Attention deficit/hyperactivity disorder
ADI-R: The Autism Diagnostic Interview-Revised Questionnaire
ADL: Everyday life skills
APA DSM-V: American Psychiatric Association, Diagnostic and Statistical Manual of Mental Disorders Diagnostic
AUC: The area under the curve
BOTM: Bruininks-Oseretsky test of Motor Proficiency
BOTMP-SF: Bruininks-Oseretsky Test of Motor Proficiency, short form
C-ABC: The Movement ABC Checklist
CAMP: Caregiver assessment of movement participation
CBCL: The child behavior checklist
CFA: Confirmatory factor analysis
ChAS-P/T: the children activity scale for parents and for teachers
CPQ: Children participation Questionnaire
CPRC: Conners Parent Rating Scale
CSAPPA: Children’s Self-Perceptions of Adequacy in and Predilection for Physical Activity
DCD: Developmental coordination disorder
DCDQ: Developmental Coordination Disorder Questionnaire
EFA: Explorative factor analysis
EYMSC: Early years movement skills checklist
GMRS: Gross Motor Rating Scale
GRADE: Grading of Recommendations Assessment, Development, and Evaluation
KBIT-2: Kaufman Brief Intelligence Test-2
KTK: Körperkoordinationstest für Kinder
MABC-2 Checklist: Movement assessment battery for children checklist – second edition
MABC-C: Movement assessment battery for children checklist
MAND: McCarron Assessment of Neuromuscular Development
MOQ-T: Motor observation questionnaire for teachers
PQ: The participation questionnaire
PSQ: the performance skills questionnaire
ROC: A receiver operating characteristic curve
SLI: Specific language impairment
TD: typically developing children
TEAF: The teacher estimation of activity form
TOMI: The test of motor impairment
VMI: Developmental test of visual-motor integration
WISC-III: Wechsler Intelligence Scale for Children – III
