# Supplementary material for: Psychometric properties of observational tools for identifying motor difficulties – a systematic review
Source: BMC Pediatr. 2019 Sep 7;19:322. doi: 10.1186/s12887-019-1657-6 (PMC6731620; doi:10.1186/s12887-019-1657-6)
Supplement: Supplementary file 1 — Search strategy. (DOCX 14 kb) [file 12887_2019_1657_MOESM1_ESM.docx]

APPENDIX 1

**ERIC, 3.11.2017, 501 studies**

((((all(developmental coordination disorder*)) OR ((all(developmental coordination disorder*)) AND pd(19940101-20171231)) OR all("developmental coordination disorder*") OR all(dyspraxia) OR (SU.EXACT("Developmental Delays") OR SU.EXACT("Motor Development") OR SU.EXACT("Learning Problems")) OR ((SU.EXACT("Developmental Delays") OR SU.EXACT("Motor Development") OR SU.EXACT("Learning Problems")) AND SU.EXACT.EXPLODE("Learning Problems")) OR all("motor problem*") OR all("motor difficult*") OR all("movement disorder*") OR (all("minimal brain dysfunction") AND pd(19940101-20171231)) OR all("motor delay")) AND pd(19940101-20171231)) AND ((SU.EXACT.EXPLODE("Screening Tests") OR (all(screening test*))) OR all(screening) OR all("screening tool") OR (SU.EXACT.EXPLODE("Questionnaires") OR all(questionnaire*)) OR (SU.EXACT.EXPLODE("Check Lists") OR all(checklist*)))) AND peer(yes) AND la.exact("English")

**PsycInfo, 6.11.2017, 155 studies**

S1 developmental coordination disorder*.mp.

S2 dyspraxia.mp. or exp DYSPRAXIA/

S3 exp Movement Disorders/ or movement disorder*.mp.

S4 motor skills disorder*.mp.

S5 motor problems.mp.

S6 probable DCD.mp.

S7 motor difficult*.mp.

S8 incoordination.mp.

S9 minimal brain dysfunction.mp.

S10 motor delay.mp.

S11 clumsy children.mp.

S12 exp SCREENING TESTS/ or screening.mp. or exp SCREENING/

S13 questionnaires/ or general health questionnaire/

S14 questionnaire*.mp. [mp=title, abstract, heading word, table of contents, key c oncepts, original title, tests & measures]

S15 checklist*.mp. [mp=title, abstract, heading word, table of contents, key concepts, original title, tests & measures]

S16 child behavior checklist/ or "checklist (testing)"/

S17 "checklist (testing)"/ or symptom checklists/

s18 1 or 2 or 3 or 4 or 5 or 6 or 7 or 8 or 9 or 10 or 11

S19 12 or 13 or 14 or 15 or 16 or 17

S20 18 and 19

**Academic Search Elite, 6.7.2017, 469 studies**

S1 DE "MOVEMENT disorders" OR DE "MOVEMENT disorders in children"

S2 "movement disorders"

S3 DE "APRAXIA"

S4 "developmental coordination disorder*"

S5 "clumsy children*"

S6 dyspraxia

S7 "motor skills disorder*"

S8 "motor problem*"

S9 "motor difficult*"

S10 incoordination

S11 S1 OR S2 OR S3 OR S4 OR S5 OR S6 OR S7 OR S8 OR S9 OR S10

S12 DE "LISTS" OR checklist*

S13 DE "CHILD Behavior Checklist"

S14 DE "QUESTIONNAIRES" OR questionnaire*

S15 (DE "MEDICAL screening" OR DE "MEDICAL screening -- Evaluation" ) OR screening

S16 S12 OR S13 OR S14 OR S15

S17 (S11) AND (S16)

S18 child*

S19 (S17) AND (S18)

**Sport discuss, 6.11.2017, 104 studies**

S19 S16 AND S17 AND S18

S18 S5 OR S6 OR S7 OR S8 OR S9 OR S10 OR S11 OR S12 OR S13 OR S14 OR S

S17 S1 OR S2 OR S3 OR S4

S16 child*

S15 motor learning difficult*

S14 clumsy child*

S13 dyspraxia

S12 apraxia

S11 motor skills disorder*

S10 motor delay

S9 DCD

S8 probable developmental coordination disorder*

S7 developmental coordination disorder*

S6 movement disorders

S5 movement disorders AND children

S4 questionnaire

S3 checklist

S2 screening OR medical screening

S1 observation educational method OR observation

**Medline, 3.11.2017, 678 studies**

#1 Motor Skills Disorders/ or developmental coordination disorder*.mp

#2 dyspraxia.mp.

#3 clumsy children*.mp

#4 motor difficult*.mp.

#5 Movement disorders/ or movement disorder*.mp.

#6 motor learning difficult*.mp.

#7 incoordination.mp.

#8 1 or 2 or 3 or 4 or 5 or 6 or 7

#9 Questionnaires/ or questionnaire*.mp.

#10 Checklist/ or checklist*.mp.

#11 Mass Screening/ or screening.mp.

#12 9 or 10 or 11

#13 8 and 12
