# Supplementary material for: Psychometric properties of observational tools for identifying motor difficulties – a systematic review
Source: BMC Pediatr. 2019 Sep 7;19:322. doi: 10.1186/s12887-019-1657-6 (PMC6731620; doi:10.1186/s12887-019-1657-6)
Supplement: Supplementary file 2 — Psychometric properties of included studies. (DOCX 57 kb) [file 12887_2019_1657_MOESM2_ESM.docx]

| **Reference** | **Measure** | **Aim** | **Country, participants, study design** | **Age** | **Validity** | **Reliability** | **Usability** | **Other measures** |
| --- | --- | --- | --- | --- | --- | --- | --- | --- |
| 1. Asunta et al. [42] | **MOQ-T-FI** | To investigate psychometric properties of the Finnish version of MOQ-T | Finland  Population (school based)  Sample 1=193  Sample 2=850 | 6-12  (S1: M 9.5)  6-9  (S2; M 7.7) | Behaves consistently with the original Dutch version  Confirmatory factor analysis: bifactor model  Correlation with MABC *r*= 0.37  Sensitivity 83% (age 6-9 86%)  Specificity 45% (age 6-9 50%)  AUC = 0.73, 95% CI 0.64 to 0.82 | Cronbach´s alpha = 0.97 | 18 items  Electronic web-based version, which is free of charge  Fast to complete: 3.3min/child | MABC-2 |
| 2.Cairney et al. [68] | **DCD-Q** | To examine psychometric properties | Canada  Population (school based) sample=523  Cross-sectional study design | 9-14 | Confirmatory factor analysis: scale was multifactorial  (fit of the hypothesised factor structure was poor)  Moderate correlations with CSAPPA (*r* = 0.38) | Cronbach’s alpha = 0.94 | 15 items perceived adequacy (7 items), predilection toward physical activity (9 items), enjoyment of physical education (3 items) | CSAPPA |
| 3.Cairney et al. [25] | **CSAPPA** | To compare the CSAPPA with a measure of motor proficiency and to evaluate the CSAPPA subscales as possible screens for DCD | Canada  Population sample  n=546  Cross-sectional study design | 9-14  (M 11.5) | AUC = 0.81, 95% CI 0.75 to 0.87  Sensitivity 86%  Specificity 47% | - | 19 items / 15-20min, scoring system easy | BOTM-SF |
| 4. Capistrano et al. [74] | **MABC Checklist** | To test the level of competition between the MABC-2 motor test and MABC-2 Checklist | Brazil  Convenient  school based sample  parents/ children=40  teachers n=16 | 7-10  (boy M 8.9)  (girl M 9.0) | Spearman correlations between DCDQ-BR and MABC-“ test *r* = 0.09  DCDQ-BR and MABC-C (classroom teacher)  *r* = -0.28  DCDQ-BR and MABC-C (PE teacher)  *r* = 0.16  DCDQ-BR and MABC-C (parent)  *r* = -0.48 | - | - | DCDQ-BR  MABC |
| 5.Caravele et al. [78] | **DCDQ-07** | To describe the adaptation to the Italian population and to test its psychometric properties | Italy  Clinical referred sample + matched controls  n=26 DCD children  n=52 TD children  Cross-sectional study design/ case-control | 5-11  (M 8.3) | Good discriminative and predictive validity (n=26 DCD children)  Sensitivity 88%  Specificity 96% | Cronbach´s alpha = 0.94  Test-retest ICC = 0.43-1 | 15 items | - |
| 6. Caravale et al. [55] | **DCDQ-Italian** | To investigate psychometric properties of DCDQ-Italian | Italy  n=698 parents  Test-retest: n=45  Concurrent val.: n=117 | 5-12  (M 7.98) | CFA supported this version consistent with the original  Sensitivity 59%  Specificity 65%  ROC = 0.62 | Cronbach´s alpha = 0.89  Test-retest  *r* = 0.88 | 15 items | MABC |
| 7. Civetta & Hillier [79] | **DCDQ** | To examine the psychometric properties of DCDQ in an Australian school population | Australia  Population sample  n=216  Cross-sectional study design | 7-8 | Factor analysis revealed discrepancies in the proposed subtest structures  Convergent validity *r* = 0.396  Sensitivity 72%  Specificity 62% | Cronbach´s alpha = 0.88 | 17 items | MABC |
| 8.De Milander et al. [93] | **DCDQ´07** | To examine the convergent validity of classification of motor difficulties | South Africa  n=410 | 5-8  (M 6.7) | Poor validity: not the best screening tool for parents  Sensitivity 23%  Specificity 91%  Kappa coefficient 0.51 -> 15% convergent validity | - | 15 items | MABC-2 |
| 9. De Milander [75] | **MABC-C** | To examine the convergent validity of the MABC-C when completed by teachers with MABC-2 test completed by Kinderkineticist | South Africa  School population sample  n=545 | 5-8 | Convergent validity: Kappa coefficient of 0.110 (11%) with small effect size (*r* = 0.125)  Sensitivity 47%  Specificity 40% | - | - | MABC-2 |
| 10. Dussart [73] | **Checklist** | To devise checklist for identifying children with suspected DCD | UK (Kent)  School population sample  3 + 7 schools  Cross-sectional study design | - | Statistically significant relationship p < 0.01 with TOMI scores  Many false positives | Control symptom (always drinking) did not show false positives | Two levels:  A) 12 items  B) 31 items | TOMI |
| 11. Engel-Yeger et al. [16] | **TEAF** | To investigate whether differences exist between children with DCD and TD children in preference to participate in leisure activities  To examine TEAF as a measure to predict participation preference | Israel  Clinical-referred sample + matched pairs  n=35 children with DCD  n=35 TD children  Cross-sectional study design/ case-control | 6─9.83 | Correlation between PAC and TEAF (*r* = -0.57, *p* < 0.0001)  TEAF mean score predicted 51% of the variance of the mean scores of seven PAC activities (*F*_1,65_ **=** 68.25, *β* = -0.71, p < 0.0001)  The findings support TEAF’s ability to differ between groups | Cronbach´s alpha = 0.64 | 10 items (5-point Likert scale) | MABC, PAC |
| 12. Faught et al. [92] | **TEAF** | To examine TEAF ability to screen children with DCD | Canada  School population sample  n=15 schools,  n=502 children, whereby 27 children (5.1%) were designated as probable DCD cases  Cross-sectional study design | 9−11 | Total TEAF scores ranging from 28 to 32 were preferred in sustaining good sensitivity (0.74, 95 % CI = 0.55-0.87 to 0.85, 95% CI= 0.68-0.94) specificity 0.46 (95% CI= 0.42-0.51)  -area under ROC curve= 0.77 (95 % CI, 0.68 - 0.86)  Correlation between TEAF and CSAPPA (*r* = -0.45, *p* < 0.001), TEAF and PQ (*r* = -0.25, *p* < 0.001) | Cronbach´s alpha = 0.98 | 10 items / 10min | BOTMP-SF  PQ  CSAPPA |
| 13. Giofre et al. [43] | **MOQ-T** | To test the validity of the Italian adaptation of the MOQ-T as a screening instrument | Italy  Study 1: n=363 children  Study 2:  n=23+23 children | M 7.7  M 8.75  M 9.7  M 10.7 | EFA and CFA analysis:  Italian version of the MOQ-T comprises two first-order factors (reflecting motor and handwriting skills), which are influenced by a second-order factor (general motor factor). The factors explained the variance of 58.26%.  The discriminatory power: children with high scores in the MOQ-T also had deficient ideomotor and praxic skills and a moderately impaired visuospatial WM | Cronbach´s alpha = 0.95 | 18 items | Ideomotor praxic abilities test  Corsi blocks test |
| 14. Girish et al. [80] | **DCDQ´07-K** | Cross-cultural validation into Kannada, Indian language | n=160 parents  (80 TD + 80 clinical group) | Clinical group, M 9.8  TD, M 11.36 | Cross-cultural val. was met  No floor and ceiling effect was found  PCA, three components explained variance of 59.29% (DCDQ-IE)  58.81% (DCDQ-K)  DCDQ (Canadian English) differed in distribution and type of items  IRT-analysis:  acceptable person reliability > 0.8, separation index > 2,  poor item reliability < 0.8, separation index <2  parents were interviewed | DCDQ-IE: Cronbach´s alpha = 0.90  DCDQ-K:  Cronbach´s alpha = 0.89  ICC.95 (0.758-0.944) |  |  |
| 15. Green et al. [69] | **DCDQ and**  **C-ABC** | To examine two questionnaires (C-ABC and DCDQ) as a method of screening children suspected of DCD  (predictive value of the questionnaires) | UK  Clinical sample: Recruited from the top of the waiting list, referred to an occupational therapy department  n=98,  DCDQ, n=97  M-ABC-C, n=75  Cross-sectional study design | 5.4-15.6 | DCDQ:  Sensitivity 93%  Specificity 19%  Positive predictive value 75%  Negative predictive value = 50%  Kappa = 0.14  C-ABC:  Sensitivity 44%  Specificity 74%  Positive predictive value 79%  Negative predictive value 37% Kappa=0.14 |  | DCDQ= 17 items  C-ABC= 5 parts | MABC |
| 16. Hay et al. [94] | **CSAPPA** | To validate the CSAPPA scale as a proxy for the BOTMP test in diagnosing DCD | Canada  Population sample  n=1 school  n=206  Cross-sectional study design | 9-16  (M 11.5) | Males:  Sensitivity 90%  Specificity 89%  Females:  Sensitivity 88%  Specificity 75% |  | 20 items / 20min | BOTMP-SF PQ |
| 17. Junaid et al. [65] | **MABC-C** | To examine the relationship between teacher´s scores (MABC-C) on physical therapists scores (MABC test) | Canada  Population sample  Children: n=103  Teacher: n = 28  Cross-sectional study design | 7.2-8.11  (M 8.0) | Sensitivity 14.3%  Specificity 97.8%  Positive predictive value = 50%  Negative predictive value = 87.9%  Correlation between MABC-C and MABC (*r* = 0.51/0.60, p > 0.001) | Test-retest reliability (n=7) 97.3% | 12 items in every four motor sections | MABC |
| 18. Kennedy-Behr et al. [81] | **DCDQ-G** | To translate and adapt DCDQ´07 into German language | Germany  Two samples: a) clinic, n=55  b) community, n=67  Cross-sectional study design | a)  5-7.11  (M 6.1)  b)  5-6.3  (M 5.6) | Both samples:  Sensitivity 52.4%  Specificity 90 %  Clinic:  Sensitivity 72.7%  Specificity 95%  Group differences (motor impaired-/non-motor impaired, U=278.5, z=-3.109, p=0.002) | Cronbach´s alpha = 0.891  Corrected item total corr. ranging from 0.418 to 0.685  AUC = 0.736 | 15 items | MABC-2 |
| 19. Loh et al. [82] | **DCDQ** | To investigate the concurrent validity of the DCDQ and MAND | Australia  Population sample  n=129  Cross-sectional study design | 9-12  (M 11.5) | Sensitivity 55 %  Specificity 74 %  DCDQ+MAND (*r* = 0.37, *p* = 0.01)  The degree of the agreement between the two measures, kappa = 0.284 | Cronbach´s alpha = 0.88  item total corr. ranging from 0.40 to 0.76 | 17 items | MAND, ADBS |
| 20. Martini et al. [83] | **DCDQ-FC** | To undertake a formal translation of DCDQ´07 and examine its psychometric properties | Canada  Population sample + clinic referred  French-Canadian children  n=84  Cross-sectional study design | 5-15  (M 9.11) | Sensitivity 47%Specificity 77%  ICC between the DCDQ-07 and MABC 0.490 | Cronbach´s alpha = 0.95  Item-total correlations (0.646-0.795)  ICC 0.465 (0.252-0.634) | 15 items | MABC-2 |
| 21. Miyachi et al. [84] | **DCDQ-J** | To investigate the degree of motor impairments in Japanese children with HFPDD | Japan  school children  n=48 boys | 6.10-15.5  (M 11.5) | HFPDD group had total DCDQ-J scores significantly lower than the standard scores of boys at the same school level  AADI-R was negatively correlated with DCDQ-J |  | 15 items | ADI-R |
| 22. Montoro et al. [85] | **DCDQ-BR** | To verify the concurrent validity with MABC2 and DCDQ-BR | Brazil  school sample  n=350 | 7-10 | Correlation between MABC-2 Total scores and DCDQ-BR:  7-8 years *r* = 0.62, p > 0.001  9-10 years *r* = 0.35, p > 0.001 |  | 15 items | MABC-2 |
| 23. Nakai et al. [59] | **DCDQ-J** | To describe the applicability of the DCDQ-J for use with a  community-based population of children in Japan | Japan  Population sample (special schools /classes excluded)  n=6330  Cross-sectional study design | 4-15 | The study employed 3-factor analysis (indicators of good ﬁt were slightly low)  Cluster analysis: 4 groups --the poor ﬁne motor group, the excellent coordination group, the excellent ﬁne motor group, and the poor coordination group | Cronbach’s alpha = 0.93  The correlation coefﬁcient between the subscales ranged from 0.59 to 0.73 | 15 items | ADHD-rating scale |
| 24. Netelenbos [63] | **GMRS** | To construct a reliable and valid unifactorial teachers´ rating scale for motor ability (gross motor skills) | Netherlands  Population sample  study 1: n=132  study 2: n=94  study 3: n=43  Test-retest: n=82  Inter-rater: n=24  Cross-sectional study design | 3-7 | Split-half coefficient = 0.98  One-dimensional structure  Correlation between stepping-stone task (*r* = -0.32)  Correlation between TGMD-L (*r* = -0.41)  correlation between MABC-B (r = 0.29, 0.18 and 0.27) -> not sig. | Cronbach’s alpha = 0.98;  Inter-rater reliability: (*r* = 0.80 and *r* = 0.98);  Test-retest: (*r* = 0.90, 0.91, 0.88, 0.79) | 20 items | TGMD-L (locomotor), MABC-B (balance); Stepping-stone task |
| 25. Nowak et al. [76] | **DCDQ´07-PL** | To present a cultural adaptation of the DCDQ´07 for the population of Polish children | Poland  normative sample  n=152 parents  clinical sample  control group n=32  KTK group n= 75  Test-retest n=50 | 5-15  normative sample M=8.66  clinical sample  M=8.89 | Sensitivity 75%  Specificity 63%  Corrected item-total correlations ranged from 0.61 to 0.73  Correlation between KTK (I = 0.726)  DCDQ-PL indicated a 63% agreement with those classified by the KTK as having DCD  Discriminate between TD children with suspected DCD | Cronbach’s alpha = 0.92  Test-retest: *r* = 0.93 |  | KTK |
| 26. Patel & Gabbard [87] | **DCDQ-Hindi** | To translate the DCDQ´07 to Hindi and test its basic psychometric properties | India  n=955  Test-retest n=60 | 5-15 | Confirmatory factor analysis showed high internal consistency  Usability was tested by field test  Item correlations ranged from 0.45 to 0.59 (except item 14; *r* = 0.24) | Cronbach’s alpha = 0.86  Moderate test-retest reliability r = 0.73 | 15 items |  |
| 27. Piek & Edwards [44] | **MABC-C** | To examine the ability of physical education and class teachers to identify children with  coordination problems using the MABC-C | Australia  Population sample  n=171  Cross-sectional study design | 9.0-10.11  (M=9.11) | *Class teachers* identified a total of 25% children identified by the performance test as clumsy. 50 % of the severe cases were identified but moderate cases only 6 %. 17 children were identified by the class teachers who were not identified as clumsy.  *Physical education* *teachers* identified 47% of clumsy children. Of these, 36% of severe cases were identified and 56% of the moderate cases. 20 children without motor problems were identified as clumsy.  Sensitivity -> low |  | 60 items, five sections, 10min | MABC  WISC - III |
| 28. Piek et al. [7] | **CBCL** | To examine the validity of the CBCL as a screening tool in identifying motor impairment | Australia  population sample  n=398  Cross-sectional study design | 3.9-14.10  (M=9.0) | Clumsy item:  Sensitivity 17%  Specificity 93%  Positive predictive value 41.7%  Negative predictive value 79.3% |  | 9 items were used | MAND |
| 29. Prado et al. [86] | **DCDQ-Brazil** | To conduct a cross-cultural adaptation of the DCDQ | Brazil  Population sample  + clinic sample (private clinics)  n=45  Cross-sectional study design | 5-12 | Gender difference in one item (runs with the same speed and form)  Wilcoxon´s test indicated that two groups (DCD and TD) were significantly different (p < 0.5)  After item, sensitivity increased from 66% to 73%, specificity 83% to 87% | ICC 0.44-1.0  Test-retest: 0.95-0.97  Cronbach’s alpha = 0.92 | 15 items (three different versions)  mean 10.44 min (SD 4.95) |  |
| 30. Ray-Kaeser et al. [58] | **DCDQ´07** | To produce a cross-cultural adaptation of the DCDQ´07 for French-Speaking Switzerland | Switzerland  n=13 parents | 5-14.4  (M=8.5) | Cognitive interview -> some items were unclear and misinterpreted. After rewording the European-French version, the DCDQ´07 is culturally appropriate |  | 15 items |  |
| 31. Rivard et al. [88] | **DCDQ´07** | To further validate DCDQ´07 and describe distributions in population-based samples | Canada  Population (school) sample  n=3070  DCD, n=122 | 8-15  (M=11.4) | Three-factor solution was found, accounting for 70.3% of the variance  There is a need for different age and sex norms  Wording of specific items (double negative) on the DCDQ may improve the validity of test scores |  | 15 items |  |
| 32. Rosenblum [60] | **ChAS-P/T** | To develop a parent and teacher questionnaire to identify children at risk for DCD | Israel  Population sample  n=216 parents  n=355 teachers  Validity sample:  n=30 children with DCD  n=30 TD children  Cross-sectional study design | 4-8  Validity sample:  5-6.5 | Factor analysis (three factors in ChAS-T and four factors in ChAS-P)  Both measures discriminate between children with and without DCD (λ = 0.60, p < 0.0001)  Correlation between ChAS-T and MABC *r* = 0.75  Correlation between ChAS-P and MABC *r* = 0.51  Correlation between ChAS-T and ChAS-P, *r* = 0.59 | Cronbach’s alpha =  0.96 - 0.94 | ChAS-T: 21 items, 5-10min/child  ChAS-P: 27 items, 5-point Likert scale, 5 min/child  Teachers reported that 21 items were easy to understand, and 6 items were difficult | MABC |
| 33. Rosenblum & Engel-Yeger [47] | **TEAF** | To test the reliability and validity of Hebrew version of TEAF questionnaire | Israel  PE teacher, n=6  Children, n=123  (TD=68, DCD=55) | 5-12  (TD, M=9.0)  (DCD, M=9.5) | Correlation between TEAF and MABC (*r* = 0.76)  One-factor solution, accounting for 82.5% of the variance  Kappa value of 0.62  Sensitivity 73%  Specificity 27% | Cronbach’s alpha = 0.97 | 10 items | MABC |
| 34. Schoemaker et al. [26] | **MABC-C** | To evaluate the psychometric properties of the MABC-C | Netherlands  Population sample + referred sample  n=120 TD,  n=64 children with DCD  Cross-sectional study design | 6-11 | Seven factors explained 73% of the variance  A significant difference was found between the mean DCD group and population sample  F (1,182) = 72.184, p < 0.001  Correlation between checklist sections and MABC test scores ranged between *r* = 0.35-0.44  Referred sample (15^th^ percentile cut-off point):  Sensitivity 65%  Specificity 66% | Cronbach’s alpha = 0.96 | 48 items | MABC |
| 35. Schoemaker et al. [4] | **DCDQ** | To investigate the psychometric properties of the DCDQ | Netherlands  Population sample  n=608  Clinic sample n=55+55  Cross-sectional study design | 4-12  (M=7.8) | Population based sample:  Sensitivity 81.6%  Specificity 89%  Referred sample:  Sensitivity 28.9%  Specificity 84%  Four factors explained 70% of the variance  A significant difference was found between the mean DCD group and population sample | Cronbach’s alpha 8y-> = 0.898  Cronbach’s alpha 4-8 years = 0.8756  (deletion of item 11, alpha will increase (0.91 and 0.89) | 17 items, 5-point scale | MABC |
| 36. Schoemaker et al. [64] | **MOQ-T** | To investigate validity of the Motor Observation Questionnaire for Teachers (MOQ-T) | Netherlands  Population sample  n=1919  Clinical-referred sample  n=182ross-sectional study design | 5─11  (clinic group M=7.7)  (TD M=7.6) | Correlations between MOQ-T and DCDQ (*r* = -0.64) and between MOQ-T and MABC (*r* = 0.57)  AUC = 0.77 (0.71-0.84)  clinic sample:  Sensitivity 80.5 %  Specificity 62%  Good discriminant validity (children in referred group and comparison *F* (1,182) = 130.442, p < 0.001 | Cronbach’s alpha = 0.95 | 18 items, 4-point Likert scale | DCDQ; MABC |
| 37. Schoemaker et al. [45] | **MABC-2 Checklist** | To investigate the validity and reliability of the MABC-2-C | Netherlands  Population sample  n=383 children  n=130 parents  Cross-sectional study design | 5-8  (M=6.9) | Five items were difficult to fill out for teachers  Six factors explained 69% of the variance  AUC = 0.67  Correlation with DCDQ-07 *r* = -0.38 and *r* = -0.36  Discriminates DCD children from TD (p < 0.001)  Sensitivity 41%  Specificity 88% | Cronbach’s alpha = 0.94 |  | DCDQ´07  MABC-2 |
| 38. Tsang et al. [61] | **CAMP** | To measure movement participation problems in children with DCD | Hong Kong  Population sample  n=465  Cross-sectional study design | 5-10 | Item separation (7.48) and child separation (3.16) were good | Reliability index for item = 0.98 | 75->35 items | MABC |
| 39. Tsang et al. [69] | **CAMP** | To investigate the psychometric properties of the CAMP, developed to measure and identify children with movement participation problems in home contexts | Hong Kong  Population sample  n=312,  n=19 children with DCD  n=293 TD children  Cross-sectional study design | 5-8 | Rasch analysis  Correlation between MABC *r* = 0.31  A significant difference was found between the mean DCD group and TD children (31.80-27.7; p < 0.05) | Test-retest ICC = 0.94 | 35 items | MABC and MABC-C |
| 40. Tseng et al. [89] | **DCDQ** | To adapt and evaluate the DCDQ for use in Chinese-speaking countries | Taiwan  Population sample  n=1082  cross-sectional study design | 6-9  (M=7.52) | Factor analyses showed this version to be compatible with the original  Sensitivity 73%  Specificity 54%  ROC=.68 | Cronbach’s alpha = 0.89  Test-retest (n=35) = 0.94 |  |  |
| 41. Van der Linde et al. [54] | **DCDDaily-Q** | To investigate the psychometric properties | Netherland  n= 218 parents  DCD children=25  TD children =193  Cross-sectional study design | 5-8  (DCD, M 6.8)  (TD, M 6.5) | 3 factors, which explained 48% of the total variance  Excellent discriminant validity  Sensitivity 88%  Specificity 92%  AUC = 0.961  Correlation for the reference group:  DCDQ -.638  MABC-2-C -.489  DCDDaily.454  MABC-2 -.360  Correlation for the DCD group:  DCDQ -.562  MABC-2-C.350  DCDDaily.037  MABC-2 -.374 | Cronbach´s alpha = 0.85 (TD) and 0.84 (DCD) | 23 items | DCDQ  MABC-2-C  MABC-2  DCDDaily |
| 42. Wilson et al. [90] | **DCDQ** | To develop a parent questionnaire to identify motor difficulties in children | Canada  n=306 | 8-15 | Concurrent validity between BOTM (*r* = 0.46)  between MABC (*r* = 0.76) |  | 17 items | BOTM  MABC |
| 43. Wilson et al. [66] | **DCDQ** | To extend the lower age range to children for DCDQ measure | Canada +UK,  Phase I: Canada,  n=5 occupational therapists  Phase II:  Canada,  n=287 children  Phase III:  Canada and England,  -phase II cases, sample in Calgary (n=87) and referred for occupational therapy in England (n=90)  Cross-sectional study design | 5-15  (M=9.0) | Sensitivity 85%  Specificity 71%  Concurrent validity between MABC (*r* = 0.55)  Concurrent validity between VMI (*r* = 0.42) | Cronbach’s alpha = 0.94 | 24 items-> 15 items | MABC  VMI |
| 44. Wright et al. [76] | **MABC-C** | To test the usefulness of the MABC-C | Singapore  Population sample  n=212  Cross-sectional study design | 7-8 | MABC-C identified 15.6% of children with DCD or risk of DCD  Teachers had difficulty in completing some items | High level of agreement between test-retest |  | MABC |
| 45. Wright & Sugden [77] | **MABC-C** | To ascertain the prevalence of DCD in Singaporean primary school children aged 6 to 9 | Singapore  Population sample  n=427  Cross-sectional study design | 6-9 | Overall prevalence:  movement problems 6.1%,  risk of DCD 10.1%  TD children 83.8% | Test-retest  (*r* = 0.50-0.94) | 5 sections | MABC |

*Note*: **ADBS**=the Australian Disruptive Behaviors Scale; **ADI-R**=the Autism Diagnostic Interview-Revised Questionnaire; **AUC**=the area under the curve; **BOTM**=Bruininks-Oseretsky test of Motor Proficiency; **BOTMP-SF**=Bruininks-Oseretsky Test of Motor Proficiency, short form; **C-ABC^*^**=The Movement ABC Checklist; **CBCL**=the Child Behavior Checklist; **CFA**=Confirmatory factor analysis; **ChAS-P/T**=the children activity scale for parents and for teachers; **CPRC**=Conners Parent Rating Scale; **CPQ**=Children participation Questionnaire**; CSAPPA**=Children´s Self-Perceptions of Adequacy in and Predilection for Physical Activity**; DCDQ**=Developmental Coordination Disorder Questionnaire; **EFA**=explorative factor analysis; **EYMSC**=Early years movement skills checklist; **GMRS**=Gross Motor Rating Scale**; KBIT-2**=Kaufman Brief Intelligence Test-2**; KTK**=Körperkoordinationstest für Kinder; **MABC-C^*^=**Movement Assessment Battery for Children Checklist; **MABC-2 Checklist**=Movement Assessment Battery for Children Checklist – Second Edition; **MAND**=McCarron Assessment of Neuromuscular Development; **MOQ-T**= Motor Observation Questionnaire for Teachers**;** **PQ**=The Participation Questionnaire**; PSQ**=the Performance Skills Questionnaire; **ROC**=a receiver operating characteristic curve; **TOMI**=the Test of Motor Impairment; **TEAF**=The teacher estimation of activity form; **TD**=typically developing children; **VMI**=Developmental test of Visual-Motor Integration; **WISC-III**=Wechsler Intelligence Scale for Children – I.

***C-ABC, MABC-C and MABC Checklist** have been used as different abbreviations from the Movement Assessment Battery for Children Checklist (Movement ABC Checklist).
